# Feline Foamy Virus Transmission in Tsushima Leopard Cats *(Prionailurus bengalensis euptilurus)* on Tsushima Island, Japan

**DOI:** 10.3390/v15040835

**Published:** 2023-03-24

**Authors:** Loai AbuEed, Isaac Makundi, Ariko Miyake, Junna Kawasaki, Chisa Minoura, Yushi Koshida, Kazuo Nishigaki

**Affiliations:** 1 Joint Graduate School of Veterinary Medicine, Yamaguchi University, 1677-1 Yoshida, Yamaguchi 753-8515, Japan; 2 College of Veterinary Medicine and Biomedical Sciences, Sokoine University of Agriculture, P.O. Box 3019, Morogoro 67125, Tanzania; 3 Faculty of Science and Engineering, Waseda University, 3-4-1 Okubo, Shinjuku-ku, Tokyo 169-8555, Japan; 4 Tsushima Wildlife Conservation Center, Saozakikoen, Kamiagata, Tsushima, Nagasaki 817-1603, Japan; 5 Conservation and Animal Welfare Trust, 642-2 Kamiagata, Tsushima, Nagasaki 817-1602, Japan

**Keywords:** foamy virus, feline, Tsushima leopard cat, domestic cat, Tsushima Island, FFV, molecular epidemiology

## Abstract

Tsushima leopard cats (TLC; *Prionailurus bengalensis euptilurus*) only inhabit Tsushima Island, Nagasaki, Japan and are critically endangered and threatened by infectious diseases. The feline foamy virus (FFV) is widely endemic in domestic cats. Therefore, its transmission from domestic cats to TLCs may threaten the TLC population. Thus, this study aimed to assess the possibility that domestic cats could transmit FFV to TLCs. Eighty-nine TLC samples were screened, and FFV was identified in seven (7.86%). To assess the FFV infection status of domestic cats, 199 domestic cats were screened; 14.07% were infected. The phylogenetic analysis revealed that the FFV partial sequence from domestic cats and TLC sequences clustered in one clade, suggesting that the two populations share the same strain. The statistical data minimally supported the association between increased infection rate and sex (*p* = 0.28), indicating that FFV transmission is not sex dependent. In domestic cats, a significant difference was observed in FFV detection in feline immunodeficiency virus (*p* = 0.002) and gammaherpesvirus1 infection statuses (*p* = 0.0001) but not in feline leukemia virus infection status (*p* = 0.21). Monitoring FFV infection in domestic cats and TLC populations is highly recommended as part of TLC surveillance and management strategies.

## 1. Introduction

The Tsushima leopard cat (TLC), also known as *Prionailurus bengalensis euptilurus,* is one of the most endangered animal species in Japan [1]. The TLC has a weight of 4.5 kg for males and 3.5 kg for females, making it a small felid. A typical cat has a long and fat tail, a long trunk, shorter-than-typical legs, and its color ranges from chestnut brown to cream with faint brown spots [2]. It only inhabits Tsushima Island in the Nagasaki Prefecture [3]. The TLC tends to be abundant in the upper region of Tsushima, known as Kamijima. Approximately 200 to 300 TLCs lived on Tsushima Island until the 1970s [4]. According to the Japan Wildlife Research Center, the number of TLCs continues to decrease at approximately 10% every 10 years. Human activity in wildcat habitats is one of the biggest threats to their survival [5]. Another important factor is infectious diseases. Disease transmission between domestic cats and TLCs have been previously documented [4,6,7,8]. Several cases of TLCs being infected with diseases commonly associated with domestic cats, including the feline immunodeficiency virus (FIV) and *Felis catus* gammaherpesvirus1 (FcaGHV1), have been reported [3,4,6]. According to a study that utilized the Geographical Information System (GIS) data, FIV infection risk in TLCs is higher in areas densely populated with domestic cats than in areas sparsely populated with the species [1]. A previous study reported the prevalence of feline leukemia virus (FeLV) infection in domestic cats but not in TLCs; however, it was demonstrated that the virus could replicate in their cells [3]. A study reported the detection of the feline foamy virus (FFV) infection in Iriomote leopard cats (*Prionailurus bengalensis iriomotensis*) in Japan [9]. A single FFV infection has been shown to be associated with potential pathological changes in domestic cats [10]. A previous study reported histopathological changes in the kidney and lung after experimental FFV inoculation [11]. Another study found that cats with FFV infection had a higher risk of developing lymphoma compared to FFV-negative cats [12]. However, coinfections with other viruses have been documented [13,14,15].

Foamy viruses (FVs) belong to the Retroviridae family, subfamily Spumaretrovirinae; they are present in cattle, horses, cats, and monkeys and are zoonotic [16,17,18,19,20]. FFVs have infected domestic cats globally, including in Asian countries [21,22], Australia [23], Germany [24], Turkey [25], and the USA [26]. FVs are complex retroviruses, similar to the lentiviral human immunodeficiency virus (HIV), and contain two terminal repeats and three main retroviral genes, *gag*, *pol*, and *env* [27,28]. All retroviruses, including FVs, replicate by first converting their RNA genome into a DNA intermediary that integrates into the host genome [29]. FFV can be divided into two distinct sequence groups (F17/951-Type and FUV-Type) in the *env* surface (SU) protein, each belonging to one neutralization group (serotype) [22]. Salivary shedding and ongoing contact between animals are speculated to be the primary routes of FV transmission, although vertical transmission through lactating cats has also been reported [10,30]. Cats may transmit the virus through bites and social contact, such as grooming [29]. Furthermore, biting and predation are major sources of transmission among the simian populations [31,32]. Cats in the USA have been found to have FFV prevalence rates ranging from 10 to 75%, with age and male sex as the risk factors [33].

The biodiversity, evolutionary dynamics, and ecology of FFV are poorly understood. Given that controlling disease in wild populations is difficult, monitoring for infectious diseases among TLC populations is an essential part of surveillance and management [6]. During the breeding season, TLCs usually have a large home range; therefore, they may contact free-roaming domestic cats. FIV prevalence among domestic cats is highest in Tsushima Island compared to other regions of Japan [1]. Most FcaGHV1 and FIV infections spread through territorial aggression and fighting according to previous epidemiological data [6]. Based on the correlation between FcaGHV1 and FIV and the identification of FIV in TLCs [1], there is a possibility that domestic cats could transmit FFV to TLCs.

To test this hypothesis, the FFV infection rate in TLCs and domestic cats was investigated, and the system to manage and protect TLCs from infectious diseases from domestic cats was evaluated. FFV infection was more frequent in domestic cats than in TLCs. The TLC sequences were identical to some domestic cat sequences over a 600-bp region of the virus. The phylogenetic analysis suggested that FFV transmission may occur between TLCs and domestic cats in Tsushima. Furthermore, monitoring infectious diseases in domestic cats will promote the conservative management of wild cats.

## 2. Materials and Methods

### 2.1. Ethics Statement

This study was approved by the Institutional Animal Care and Use Committee of Yamaguchi University (identification code 2017/315). Animal studies were conducted in accordance with the guidelines for the Care and Use of Laboratory Animals of the Ministry of Education, Culture, Sports, Science, and Technology, Japan.

### 2.2. Study Location

**Figure 1:**
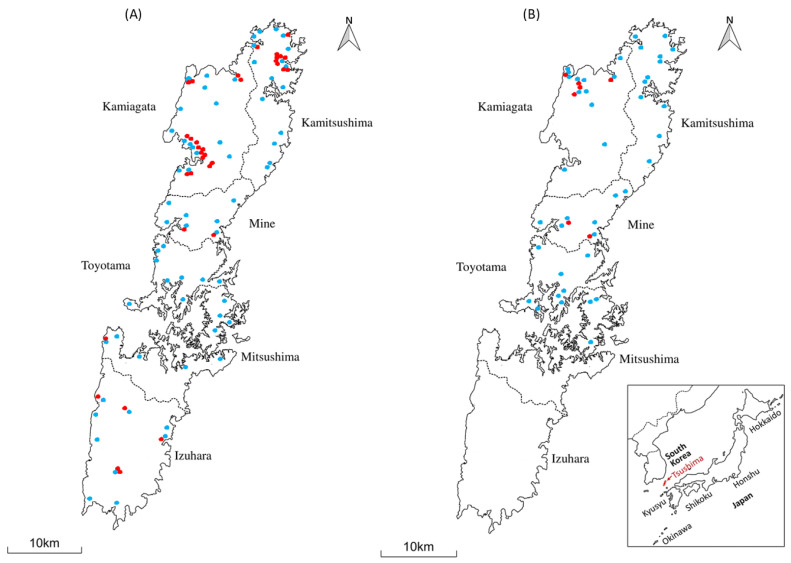
Maps of Tsushima Island and sample collection sites. (**A**) Map of sample collection sites for domestic cats. (**B**) Map of sample collection sites for wild cats. Blue dots indicate sample locations. Red dots represent positive samples.

Tsushima (34°05′–34°42′ N, 129°10′–129°30′ E) is an island in the Japanese archipelago with a surface area of 708.6 km^2^. It lies 50 km from Busan, the Korean peninsula, and 138 km from Kyushu Island, Japan. There are two main islands on Tsushima Island: the northern and southern Tsushima islands called Kamijima and Shimojima, respectively. There are four boroughs in Kamijima (Kamitsushima, Kamiagata, Mine, and Toyotama) and two in Shimojima (Mitsushima and Izuhara) (Figure 1).

### 2.3. Sample Collection

In total, 89 TLC (60 blood and 29 spleen) samples were obtained from Tsushima Island (Figure 1B) between 1999 and 2020 [3]. TLC samples were collected by the Kyushu Regional Environment Office (Tsushima Wildlife Conservation Center, Tsushima, Nagasaki, Japan), Ministry of Environment, Japan. In most cases, the cats were killed by vehicles. The Tsushima Animal Medical Center, a non-profit animal hospital on the island, donated 199 domestic cat blood samples [3]; the domestic cats brought to the center between 2009 and 2015 included both indoor-only and free-roaming cats. Blood samples and spleens were stored at −80 °C, and working samples were stored at −30 °C. Figure 1A shows the sample collection site for domestic cats on Tsushima Island.

Blood samples were screened for FeLV and FIV infections using the SNAP FeLV/FIV Combo Kit (IDEXX Laboratories Inc., Westbrook, ME, USA). Total DNA was extracted from whole blood samples using the Dr. GenTLE System (Takara Bio Inc., Kyoto, Japan) and DNAzol reagent (Life Technologies Japan, Tokyo, Japan) according to the manufacturer’s instructions. DNA was extracted from the spleen using a commercial kit (DNeasy^®^ Blood & Tissue Kit; QIAGEN, Hilden, Germany). Conventional PCR for feline glyceraldehyde-3-phosphate dehydrogenase (GAPDH) was conducted as previously described to confirm the presence of amplifiable template DNA [34].

### 2.4. PCR Amplification and Sequencing

The nested-PCR amplification method for FFV was conducted as described previously [14] using the primers listed in Table 1. Primers were synthesized by Fasmac Co., Ltd., Kanagawa, Japan and dissolved in ultra-pure water (PCR grade) to a concentration of 100.0 pmol/µL, from which working dilutions were made to a final concentration of 10.0 pmol/µL and stored at −30 °C until use.

The total volume of the reaction mixture used for each nested PCR was 24.0 μL. The nested PCR reaction mixture contained 12.5 μL of KOD One ^TM^ PCR master Mix (TOYOBO Bio Inc., Osaka, Japan), 8.5 μL of ultra-pure water (PCR grade), and 1.5 μL of each of the forward and reverse primer working stock. In the first PCR round (nested PCR 1), 1.0 μL template DNA was added, spun quickly, and placed on a thermocycler with 30 cycles of denaturation at 98 °C for 10 s, annealing at 63 °C for 5 s, and extension at 68 °C for 8 s. For the second PCR round (nested PCR 2), 1 μL of the PCR 1 product was added, quickly spun, and placed in a thermocycler with 30 cycles of denaturation at 98 °C for 10 s, annealing at 63 °C for 5 s, and extension at 68 °C for 1 s. PCR amplification products were further identified using electrophoresis with 1% agarose gels and purified using a FastGene Gel/PCR extraction kit (Nippon Genetics Co., Ltd., Tokyo, Japan). DNA sequencing by Fasmac Co., Ltd., Kanagawa, Japan, was performed on the second PCR product in both directions. UGENE 45.0 software was used to visualize and analyze the sequences, and GeneTyx (Software Development Co., Tokyo, Japan) and NCBI BLAST programs were used to compare the 600 bp SU region sequences with other FFV SU region sequences.

### 2.5. Multiple Sequence Alignment and Phylogenetic Analysis

The SU region nucleotide sequences for the samples and international FFV isolates, which were retrieved from GenBank, were aligned using ClustalW [35]. Phylogenetic analyses were performed using two different software packages. The most suitable method for phylogenetic analysis at the nucleic acid level was determined using the jModel-test [36]. MEGA6 was used for phylogenetic analysis. The phylogenetic tree was constructed using the maximum likelihood (Kimura 2-parameter) method for nucleic acids [35]. A total of 1000 bootstrap replicates were used to construct the phylogenetic tree.

### 2.6. Statistical Analyses

All statistical analyses were performed using the Minitab Statistical program (Minitab version 18, Minitab Inc., Shanghai, China, 2018). The odds ratios (ORs), 95% confidence intervals (95% CIs), and *p*-values were calculated to analyze the risk factors for FFV and FFV co-infection; *p* values < 0.05 were considered statistically significant.

## 3. Results

### 3.1. Prevalence and Demographics

The creation of amplicons relies on the primer that amplifies the sequence of the partial *env* gene (SU region). Therefore, nested PCR primers were obtained from a previous study [14]. The nested PCR method successfully amplified the SU region in seven TLC samples indicating an overall FFV detection frequency of 7.86% in TLCs (Table 2). These seven FFV-positive TLCs originated from Kamijima and comprised four males and three females.

Given that domestic cats are the primary natural hosts of FFV, their FFV infection status was investigated to better understand TLC management. In total, 28 of the 199 (14.07%) domestic cats were positive for FFV according to the nested PCR test (Table 2). The characteristics of the domestic cats tested in this study are presented in Table 3. No significant difference was observed in the FFV-positive sample (FFV infection rate) between Kamijima and Shimojima (Table 4). Of the 35 FFV-positive samples for domestic cats and TLCs, 29 (82.85%) were obtained from Kamitsushima and Kamiagata (Figure 1A,B).

### 3.2. FFV Co-Infection with FIV, FeLV, and FcaGHV1

To study the dynamics of retrovirus infection in TLCs, FFV co-infection with FeLV, FIV, and FcaGHV1 in the 199 domestic cats, which had previously been tested for FeLV, FIV, and FcaGHV1, was investigated (Table 3). FIV and FcaGHV1 infection statuses were significantly associated with FFV detection (Table 4). In contrast, FeLV infection status was not associated with FFV detection. Furthermore, no sex difference was observed for single infections of FFV and FeLV in the domestic cat samples (Table 4 and Table 5). FIV and FcaGHV1 had higher infection rates in males than females (Table 5). In contrast, the FFV and FeLV coinfection rates in males were not significantly different from those in females.

### 3.3. Nucleotide Similarity with SU Region for FFV

**Figure 2:**
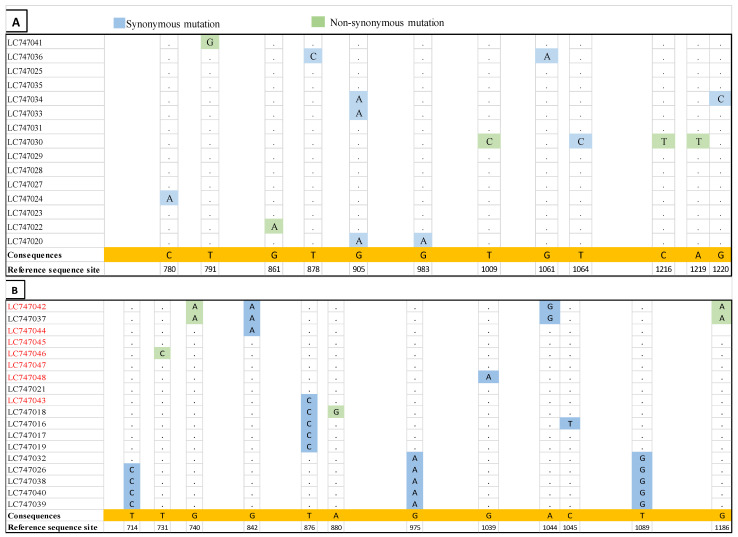
Comparison of the nucleic acid sequence of the SU region. (**A**) Multiple sequence alignment of the SU region for F17/951-Type. (**B**) Multiple sequence alignment of the SU region for FUV-Type. Dots indicate identical consequences. Black and red color labels indicate domestic cat and TLC samples, respectively.

In total, 35 FFV-positive samples (7 TLCs and 28 domestic cats) were sequenced; however, two domestic cat samples were lost. Nucleotide sequences of the partial *env* gene (SU region) were determined. Two distinct *env* genotypes (F17/951-Type and FUV-Type) were identified with a nucleotide similarity ranging from 98 to 100% and 82 to 85% within and between genotypes, respectively (Figure 2). A total of 12 mutations (5 non-synonymous and 7 synonymous) were identified in 15 samples (F17/951-Type) (Figure 2A), and 12 (4 non-synonymous and 8 synonymous) in 18 samples (FUV-Type) (Figure 2B). The FUV and F17/951 types are distributed along the Tsushima Island (Figure S1). The nucleotide differences may appear as a logical result because the amplification target in this study was the SU region, which is considered a hypervariable region.

### 3.4. Phylogenetic Analysis of FFV Based on SU Region

**Figure 3:**
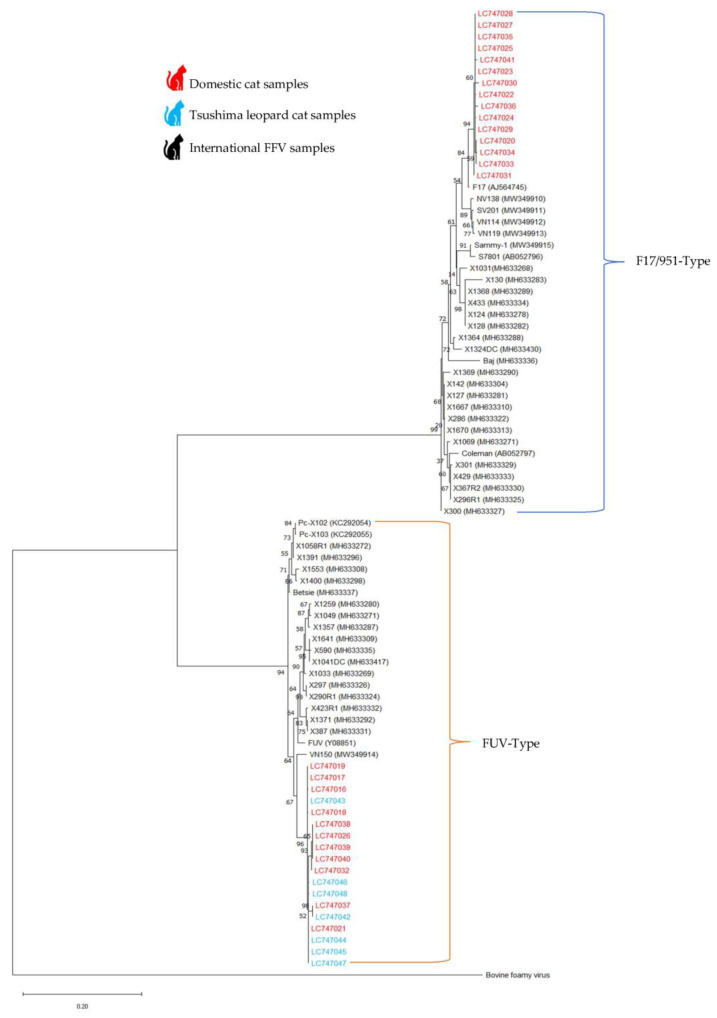
Maximum likelihood tree for SU region nucleic acid sequence. The percentage of replicate trees in which the associated taxa clustered in the bootstrap test (1000 replicates) are shown next to the branches. The tree is drawn to scale with branch lengths in the same units as those of the evolutionary distances used to infer the phylogenetic tree. The evolutionary distances were computed using the Kimura 2-parameter method and are in the units of the number of base differences per site. This analysis involved 84 nucleotide sequences.

A total of 33 FFV sequences and 51 international FFV sequences retrieved from the GenBank database were used to construct a phylogenetic tree based on nucleic acids (Figure 3). Phylogenetic analysis demonstrated that 15 FFV-positive domestic cat samples belonged to the F17-Type and 18 samples (11 domestic cat and 7 TLC samples) belonged to the FUV-Type. Evidence of FFV transmission on Tsushima Island is strong, although some subclades indicate region-specific viral populations. FFV isolates from domestic cats were interspersed throughout the tree; in contrast, TLC FFV isolates were confined to one clade.

### 3.5. Nucleotide Sequence Accession Numbers

The partial FFV nucleotide sequences obtained in this study were deposited in the DDBJ, GenBank, and EMBL databases under accession numbers LC747016–LC747048.

## 4. Discussion

This study is the first molecular survey and genetic analysis of FFV in TLCs (*Prionailurus bengalensis euptilurus*). FFV detection in felids other than domestic cats, which are its natural hosts, suggests that FFV can be transmitted to other felines. There is no “gold standard” diagnostic test for FFV, and most previous studies have used qPCR assays. FFV may not be detected in spleen samples in some cases, even if other tissues or fluids (such as blood or saliva) test positive, depending on the stage of infection [10]. According to a previous study, enzyme-linked immunoassay (ELISA) and PCR tests had similar sensitivities; however, ELISA had a higher specificity [16]. As a result, it is possible that the assays in this study do not detect all FFV positive cases in either the domestic cats or TLC populations.

Previous studies have demonstrated FIV and FcaGHV1 transmission in TLC [1,6]. The phylogenetic analysis of retrovirus-positive individuals supports the transmission of FIV and FcaGHV1 from domestic cats to TLC on Tsushima Island. Therefore, FFV was included in this survey because TLCs only inhabit this region, and FFV transmission from domestic cats to TLCs may affect management strategies for protecting this endangered species.

The prevalence of FFV in domestic cats and TLCs in Tsushima was 14.8% (domestic cats, 6.48%; TLCs, 7.7%), similar to the previously reported prevalence, which ranged from 8% to 80%, based on geographic location [16,24]. Thus, domestic cats are more likely to be infected with FFV than TLCs, and FFV may be a cross-species transmission infection. Furthermore, FFV transmission may be possible in other cat species. No significant difference was observed in the FFV infection rate between Kamijima and Shimojima (ORs, 1.01; 95% CI, 0.38–2.67). The identification of FFV-positive samples from Kamijima and Shimojima indicated that FFV is widely spread across free-ranging populations. Although no FFV-positive TLC was detected in Shimojima, FFV infection of TLC may spread there in the future as it has been confirmed to be present in the area in recent years (Figure 1B).

FFV has been suggested to be pathogenic through co-infection with retroviruses [30]. FFV co-infection with FeLV or FIV has been reported to increase FeLV proviral load and detection rate [37,38]; previous studies have shown that non-regenerative anemia and T-cell lymphoma may occur [14]. Therefore, the relationship between FFV infection and other infections (FeLV, FIV, and FcaGHV1) were analyzed. FIV- and FcaGHV-positive individuals had a higher FFV infection rate than the negative individuals. In contrast, FeLV-positive individuals had a lower FFV infection rate than negative individuals (OR, 0.39; 95% CI, 0.08–1.74). Therefore, FFV may spread horizontally by bites, similar to FIV and FcaGHV1. Among the 199 domestic cat samples tested, 183 were strays or cats housed outdoors, whereas 16 were completely housed cats, suggesting that the population bias affected the study outcome. Most sampled cats died from traffic accidents and were discovered by chance; therefore, the relationship between the co-infection of these viruses and pathogenicity was unknown. In addition, these infectious diseases may have caused the death of individuals living in isolated environments.

Previous studies have demonstrated that the male sex is a significant risk factor for FcaGHV1 infection [34,39,40]. In addition, the FIV infection rate in males has been reported to be higher than that in females [6]. The statistical data from this study minimally supported the association between the increased single infection rate of FFV and FeLV and sex, indicating that FFV is readily transmitted between sexes. Additionally, previous reports have indicated that sex may not be a risk factor for FFV infection [29]. FIV and FcaGHV1 had higher infection rates in males, consistent with previous findings [41]. Regarding the sex difference in co-infection rates between FFV and other retroviruses, males were more likely to have a co-infection with FFV and FIV than females (OR, 3.30; 95% CI, 1.29–8.43). Furthermore, the co-infection rate of FFV and FcaGHV1 was higher in males (OR, 6.02; 95% CI, 1.62–22.34), suggesting that horizontal transmission by bite is the main route of infection in domestic male cats, similar to FIV and FcaGHV1, whereas FFV may be transmitted in domestic female cats via a different infection route (vertical transmission, etc.). No significant sex-related difference was observed in the co-infection rate between FFV and FeLV (OR, 1.26; 95% CI, 0.07–20.50). Therefore, it is unlikely that FFV would be transmitted through a prolonged contact route.

According to phylogenetic analysis, TLCs and domestic cats are hosts for the same virus. Phylogenetic analysis demonstrated that all TLC isolates belonged to one clade; however, domestic cat isolates clustered throughout the phylogenetic tree, basal to or within the TLC dominant clade (Figure 3). Additionally, F17/951-Type spreads between domestic cats, while FUV-Type infects both domestic cats and TLCs. A previous study reported that domestic cats are the main host of FFV [37], and that there is a high possibility of FFV transmission from domestic cats to TLCs. Previous studies have demonstrated the viral transmission of FIV and FcaGHV1 in TLCs [1,6]. Phylogenetic analysis of these retrovirus-positive individuals supports the interspecific transmission of FIV and FcaGHV1 from domestic cats to TLCs [1]. According to recent reports, wild cats are increasingly preying on domestic animals near populated areas, suggesting an increase in the viral spillover from domestic cats to wild cats [42,43].

Phylogenetic analysis of the SU region indicated two distinct clades denoting the two SU *env* genotypes (F17-Type and FUV-Type; Figure 3). Nucleotide sequence similarity within and between genotypes ranged from 98 to 100% and 83 to 85%, respectively. Similar mutations between the isolates (LC747042 and LC747037) support our suggestion that the FFV virus is transmitted between the domestic cat and the TLC. In addition, random mutations have been found in some isolates (such as LC747041) that may occur after the infection of cats. Both SU *env* genotypes were isolated from domestic cats and obtained from Kamijima and Shimojima. This suggests a synergistic interaction between both genotypes, which are adapted to domestic cats and regularly co-circulate. Both genotypes were highly prevalent in domestic cats, suggesting that they are well-adapted despite the large quantity of amino acid variability in this SU region. Recent studies have reported two unique *env* SFV subtypes that are consistent with this observation in non-human primates [44,45]. In domestic cats, serotype-specific PCR, sequencing, and neutralizing assays have been used to identify these two genotypes [23]. In addition, a previous study reported the existence of the F17-Type in Iriomote cats on the Yaeyama Islands of Japan [46]. However, there are no available data about the existence of the FUV-Type in Japan; therefore, this study is the first to report the existence of the FUV-Type on Tsushima Island. Both SU *env* genotypes were isolated from domestic cats, and one genotype (FUV-Type) was isolated from TLCs, supporting the speculation that the virus was moved to Tsushima. Additionally, further research on the transmission routes and co-infections is required for the revision of this management regime.

## 5. Conclusions

The study findings demonstrated that TLCs were infected with FFV, in addition to previously reported diseases, which may be originating from domestic cats. Furthermore, the infection rate of FFV in TLCs was approximately 7.86% indicating that it was more widespread than FcaGHV1. The regional distribution of positive samples, co-infection trends, and previous findings support the hypothesis that FFV may be transmitted interspecies between domestic cats and TLCs. Consequently, domestic cats and TLCs may have a closer relationship than previously speculated, posing a new challenge for the management and protection of endangered TLCs.

Considering the infectious diseases that originate from domestic cats, based on previous research and the FFV infection rate in TLCs in this study, various measures are necessary for TLC conservation. Specifically, to minimize the interaction between TLCs and domestic cats, stray domestic cats in Tsushima should be eliminated, and households should be advised to keep their cats indoors and ensure their thorough vaccination. In addition to regular testing for FeLV and FIV, testing for FFV infection should be added as a new index to investigate the spread of infectious diseases.

## Figures and Tables

**Table 1:** Primer used in nested-PCR amplification. The expected amplicon size after the second PCR is 600 nucleotides.

Target Gene	Name	Sense	Primer Sequence (5′-3′)	Reference
Envelope	env-f2	1st forword	GCTACTTCTACTAGAATAATGTTTTGGATA	[14]
	env-r2	1st reverse	AGCCACAGTAGTAATTGCATGGCCAGGCC	[14]
	env-f3	2nd forward	GCTTTCAAAAATATGGACATTGTTATGTTA	[14]
	env-r3	2nd reverse	GTTTCTCCAAAATCTGCAAGCATATGGATG	[14]

**Table 2:** FFV detected in feline DNA sample by nested PCR.

Host Species	No. of Samples	No. of FFV Positive	% Positive
Tsushima leopard cat	89	7	7.86
Domestic cat	199	28	14.07

**Table 3:** Prevalence of FFV among domestic cat samples.

		FFV Status			
Variable	Categories	Positive	Negative	Total	% Positive
Sex	Male	15	73	88	17.04
	Female	13	98	111	11.71
Location	Kamijima	22	134	156	14.10
	Shimojima	6	37	43	13.95
FIV ^1^	Positive	23	61	84	27.38
	Negative	12	103	115	10.43
FeLV ^2^	Positive	2	22	24	8.33
	Negative	33	142	175	18.85
FcaGHV1 ^1^	Positive	14	11	25	56
	Negative	21	153	174	12.06

^1^ Cited from reference number 8; ^2^ Cited from reference number 2.

**Table 4:** Univariable logistic regression analysis to evaluate the association between variables and FFV detection in domestic cats.

Variables	Categories	Z Statistic	Odds Ratio	95% CI ^1^	*p* Value
Location	Kamijima vs. Shimojima	0.02	1.01	0.38–2.67	0.98
Sex	Male vs. female	1.06	1.54	0.69–3.45	0.284
FIV	Positive vs. negative	3	3.23	1.50–6.96	0.002
FeLV	Positive vs. negative	1.22	0.391	0.08–1.74	0.21
FcaGHV1	Positive vs. negative	4.78	9.27	3.72–23.08	0.0001

^1^ Confidence intervals; CI, Confidence interval.

**Table 5:** Evaluation of the association between sex and another feline virus detection in domestic cats.

Variable	No. of Positive Samples	Male	Female	% Positive	Z Statistic	Odds Ratio	95% CI ^1^	*p* Value
FIV	84	51	33	42.2	3.94	3.25	1.81–5.86	0.0001
FeLV	24	12	12	12	0.60	1.30	0.55–3.06	0.54
FcaGHV1	25	17	8	12.2	2.47	3.08	1.26–7.53	0.013
FeLV + FFV	2	1	1	1	0.16	1.26	0.07–20.50	0.86
FIV + FFV	23	16	7	11.6	2.49	3.30	1.29–8.43	0.012
FcaGHV1 + FFV	14	11	3	7.03	2.68	6.02	1.62–22.34	0.007

^1^ Confidence intervals; CI, Confidence interval.

## Data Availability

Data are available on request to the corresponding author.

## Supplementary Materials

The following supporting information can be downloaded at: https://www.mdpi.com/article/10.3390/v15040835/s1. Figure S1. Maps of Tsushima Island and positive sample sites.

## References

[1] Makundi I., Koshida Y., Endo Y., Nishigaki K. (2018). Identification of Felis Catus Gammaherpesvirus 1 in Tsushima Leopard Cats (Prionailurus Bengalensis Euptilurus) on Tsushima Island, Japan. Viruses.

[2] Saitoh T., Kaji K., Izawa M., Yamada F. (2015). Conservation and Management of Terrestrial Mammals in Japan: Its Organizational System and Practices. Therya.

[3] Makundi I., Koshida Y., Kuse K., Hiratsuka T., Ito J., Baba T., Watanabe S., Kawamura M., Odahara Y., Miyake A. (2017). Epidemiologic Survey of Feline Leukemia Virus in Domestic Cats on Tsushima Island, Japan: Management Strategy for Tsushima Leopard Cats. J. Vet. Diagn. Investig..

[4] Tateno M., Nishio T., Matsuo T., Sakuma M., Nakanishi N., Izawa M., Asari Y., Okamura M., Miyama T.S., Setoguchi A. (2013). Epidemiological Survey of Tick-Borne Protozoal Infection in Iriomote Cats and Tsushima Leopard Cats in Japan. J. Vet. Med. Sci..

[5] Mitani N., Mihara S., Ishii N., Koike H. (2009). Clues to the Cause of the Tsushima Leopard Cat (Prionailurus Bengalensis Euptilura) Decline from Isotopic Measurements in Three Species of Carnivora. Ecol. Res..

[6] Hayama S., Yamamoto H., Nakanishi S., Hiyama T., Murayama A., Mori H., Sugitani A., Fujiwara S. (2010). Risk Analysis of Feline Immunodeficiency Virus Infection in Tsushima Leopard Cats (Prionailurus Bengalensis Euptilurus) and Domestic Cats Using a Geographic Information System. J. Vet. Med. Sci..

[7] Tateno M., Nishio T., Sakuma M., Nakanishi N., Izawa M., Asari Y., Okamura M., Maruyama S., Miyama T.S., Setoguchi A. (2013). Molecular Epidemiologic Survey of Bartonella, Ehrlichia, and Anaplasma Infections in Japanese Iriomote and Tsushima Leopard Cats. J. Wildl. Dis..

[8] Nishimura Y., Goto Y., Yoneda K., Endo Y., Mizuno T., Hamachi M., Maruyama H., Kinoshita H., Koga S., Komori M. (1999). Interspecies Transmission of Feline Immunodeficiency Virus from the Domestic Cat to the Tsushima Cat (Felis Bengalensis Euptilura) in the Wild. J. Virol..

[9] Sumiyoshi A., Kitao K., Miyazawa T. (2022). Genetic and Biological Characterization of Feline Foamy Virus Isolated from a Leopard Cat (Prionailurus Bengalensis) in Vietnam. J. Vet. Med. Sci..

[10] Ledesma-Feliciano C., Troyer R.M., Zheng X., Miller C., Cianciolo R., Bordicchia M., Dannemiller N., Gagne R., Beatty J., Quimby J. (2019). Feline Foamy Virus Infection: Characterization of Experimental Infection and Prevalence of Natural Infection in Domestic Cats with and without Chronic Kidney Disease. Viruses.

[11] Alke A., Schwantes A., Zemba M., Flügel R.M., Löchelt M. (2000). Characterization of the Humoral Immune Response and Virus Replication in Cats Experimentally Infected with Feline Foamy Virus. Virology.

[12] Court E.A., Watson A.D.J., Peaston A.E. (1997). Retrospective Study of 60 Cases of Feline Lymphosarcoma. Aust. Vet. J..

[13] Alais S., Pasquier A., Jegado B., Journo C., Rua R., Gessain A., Tobaly-Tapiero J., Lacoste R., Turpin J., Mahieux R. (2018). STLV-1 Co-Infection Is Correlated with an Increased SFV Proviral Load in the Peripheral Blood of SFV/STLV-1 Naturally Infected Non-Human Primates. PLoS Negl. Trop. Dis..

[14] Cavalcante L.T., Muniz C.P., Jia H., Augusto A.M., Troccoli F., Medeiros S.D.O., Dias C.G., Switzer W.M., Soares M.A., Santos A.F. (2018). Clinical and Molecular Features of Feline Foamy Virus and Feline Leukemia Virus Co-Infection in Naturally-Infected Cats. Viruses.

[15] Switzer W.M., Garcia A.D., Yang C., Wright A., Kalish M.L., Folks T.M., Heneine W. (2008). Coinfection with HIV-1 and Simian Foamy Virus in West Central Africans. J. Infect. Dis..

[16] Dannemiller N.G., Kechejian S., Kraberger S., Logan K., Alldredge M., Crooks K.R., VandeWoude S., Carver S. (2020). Diagnostic Uncertainty and the Epidemiology of Feline Foamy Virus in Pumas (*Puma concolor*). Sci. Rep..

[17] Mekata H., Okagawa T., Konnai S., Miyazawa T. (2021). Molecular Epidemiology and Whole-Genome Analysis of Bovine Foamy Virus in Japan. Viruses.

[18] Hooks J.J., Gibbs C.J. (1975). The Foamy Viruses. Bacteriol. Rev..

[19] Khan A.S., Bodem J., Buseyne F., Gessain A., Johnson W., Kuhn J.H., Kuzmak J., Lindemann D., Linial M.L., Löchelt M. (2018). Spumaretroviruses: Updated Taxonomy and Nomenclature. Virology.

[20] Tobaly-Tapiero J., Bittoun P., Neves M., Guillemin M.-C., Lecellier C.-H., Puvion-Dutilleul F., Gicquel B., Zientara S., Giron M.-L., de Thé H. (2000). Isolation and Characterization of an Equine Foamy Virus. J. Virol..

[21] Pinto-Santini D.M., Stenbak C.R., Linial M.L. (2017). Foamy Virus Zoonotic Infections. Retrovirology.

[22] Phung H.T.T., Ikeda Y., Miyazawa T., Nakamura K., Mochizuki M., Izumiya Y., Sato E., Nishimura Y., Tohya Y., Takahashi E. (2001). Genetic Analyses of Feline Foamy Virus Isolates from Domestic and Wild Feline Species in Geographically Distinct Areas. Virus Res..

[23] Winkler I.G., Flügel R.M., Löchelt M., Flower R.L.P. (1998). Detection and Molecular Characterisation of Feline Foamy Virus Serotypes in Naturally Infected Cats. Virology.

[24] Bleiholder A., Mühle M., Hechler T., Bevins S., Denner J., Löchelt M. (2011). Pattern of Seroreactivity against Feline Foamy Virus Proteins in Domestic Cats from Germany. Vet. Immunol. Immunopathol..

[25] Koc B.T., Oğuzoğlu T.Ç. (2019). First Report on the Prevalence and Genetic Relatedness of Feline Foamy Virus (FFV) from Turkish Domestic Cats. Virus Res..

[26] Kechejian S.R., Dannemiller N., Kraberger S., Ledesma-Feliciano C., Malmberg J., Roelke Parker M., Cunningham M., McBride R., Riley S.P.D., Vickers W.T. (2019). Feline Foamy Virus Is Highly Prevalent in Free-Ranging *Puma Concolor* from Colorado, Florida and Southern California. Viruses.

[27] Kehl T., Tan J., Materniak M. (2013). Non-Simian Foamy Viruses: Molecular Virology, Tropism and Prevalence and Zoonotic/Interspecies Transmission. Viruses.

[28] Roy J., Rudolph W., Juretzek T., Gärtner K., Bock M., Herchenröder O., Lindemann D., Heinkelein M., Rethwilm A. (2003). Feline Foamy Virus Genome and Replication Strategy. J. Virol..

[29] Kraberger S., Fountain-Jones N.M., Gagne R.B., Malmberg J., Dannemiller N.G., Logan K., Alldredge M., Varsani A., Crooks K.R., Craft M. (2020). Frequent Cross-Species Transmissions of Foamy Virus between Domestic and Wild Felids. Virus Evol..

[30] Romen F., Backes P., Materniak M., Sting R., Vahlenkamp T.W., Riebe R., Pawlita M., Kuzmak J., Löchelt M. (2007). Serological Detection Systems for Identification of Cows Shedding Bovine Foamy Virus via Milk. Virology.

[31] Mouinga-Ondémé A., Caron M., Nkoghé D., Telfer P., Marx P., Saïb A., Leroy E., Gonzalez J.-P., Gessain A., Kazanji M. (2012). Cross-Species Transmission of Simian Foamy Virus to Humans in Rural Gabon, Central Africa. J. Virol..

[32] Buseyne F., Betsem E., Montange T., Njouom R., Bilounga Ndongo C., Hermine O., Gessain A. (2018). Clinical Signs and Blood Test Results among Humans Infected with Zoonotic Simian Foamy Virus: A Case-Control Study. J. Infect. Dis..

[33] Kechejian S., Dannemiller N., Kraberger S., Ledesma Feliciano C., Löchelt M., Carver S., VandeWoude S. (2019). Feline Foamy Virus Seroprevalence and Demographic Risk Factors in Stray Domestic Cat Populations in Colorado, Southern California and Florida, USA. J. Feline Med. Surg. Open Rep..

[34] Beatty J.A., Troyer R.M., Carver S., Barrs V.R., Espinasse F., Conradi O., Stutzman-Rodriguez K., Chan C.C., Tasker S., Lappin M.R. (2014). Felis Catus Gammaherpesvirus 1; a Widely Endemic Potential Pathogen of Domestic Cats. Virology.

[35] Tamura K., Stecher G., Peterson D., Filipski A., Kumar S. (2013). MEGA6: Molecular Evolutionary Genetics Analysis Version 6.0. Mol. Biol. Evol..

[36] Darriba D., Taboada G.L., Doallo R., Posada D. (2012). JModelTest 2: More Models, New Heuristics and Parallel Computing. Nat. Methods.

[37] Winkler I.G., Lochelt M., Flower R.L.P. (1999). Epidemiology of Feline Foamy Virus and Feline Immunodeficiency Virus Infections in Domestic and Feral Cats: A Seroepidemiological Study. J. Clin. Microbiol..

[38] De Miranda L.H.M., Meli M., Conceição-Silva F., Novacco M., Menezes R.C., Pereira S.A., Sugiarto S., dos Reis É.G., Gremião I.D.F., Hofmann-Lehmann R. (2018). Co-Infection with Feline Retrovirus Is Related to Changes in Immunological Parameters of Cats with Sporotrichosis. PLoS ONE.

[39] McLuckie A., Tasker S., Dhand N.K., Spencer S., Beatty J.A. (2016). High Prevalence of Felis Catus Gammaherpesvirus 1 Infection in Haemoplasma-Infected Cats Supports Co-Transmission. Vet. J..

[40] Ertl R., Korb M., Langbein-Detsch I., Klein D. (2015). Prevalence and Risk Factors of Gammaherpesvirus Infection in Domestic Cats in Central Europe. Virol. J..

[41] Stutzman-Rodriguez K., Rovnak J., VandeWoude S., Troyer R.M. (2016). Domestic Cats Seropositive for Felis Catus Gammaherpesvirus 1 Are Often QPCR Negative. Virology.

[42] Moss W.E., Alldredge M.W., Logan K.A., Pauli J.N. (2016). Human Expansion Precipitates Niche Expansion for an Opportunistic Apex Predator (*Puma concolor*). Sci. Rep..

[43] Chiu E.S., Kraberger S., Cunningham M., Cusack L., Roelke M., VandeWoude S. (2019). Multiple Introductions of Domestic Cat Feline Leukemia Virus in Endangered Florida Panthers. Emerg. Infect. Dis..

[44] Richard L., Rua R., Betsem E., Mouinga-Ondémé A., Kazanji M., Leroy E., Njouom R., Buseyne F., Afonso P.V., Gessain A. (2015). Cocirculation of Two Env Molecular Variants, of Possible Recombinant Origin, in Gorilla and Chimpanzee Simian Foamy Virus Strains from Central Africa. J. Virol..

[45] Lambert C., Couteaudier M., Gouzil J., Richard L., Montange T., Betsem E., Rua R., Tobaly-Tapiero J., Lindemann D., Njouom R. (2018). Potent Neutralizing Antibodies in Humans Infected with Zoonotic Simian Foamy Viruses Target Conserved Epitopes Located in the Dimorphic Domain of the Surface Envelope Protein. PLoS Pathog..

[46] Mochizuki M., Akuzawa M., Nagatomo H. (1990). Serological Survey of the Iriomote Cat (*Felis iriomotensis*) in Japan. J. Wildl. Dis..

