# Supplementary material for: Feline Foamy Virus Transmission in Tsushima Leopard Cats (Prionailurus bengalensis euptilurus) on Tsushima Island, Japan
Source: Viruses. 2023 Mar 24;15(4):835. doi: 10.3390/v15040835 (PMC10146696; doi:10.3390/v15040835)
Supplement: Supplementary file 1 [file viruses-15-00835-s001.zip › viruses-2240541-supplementary.pdf]

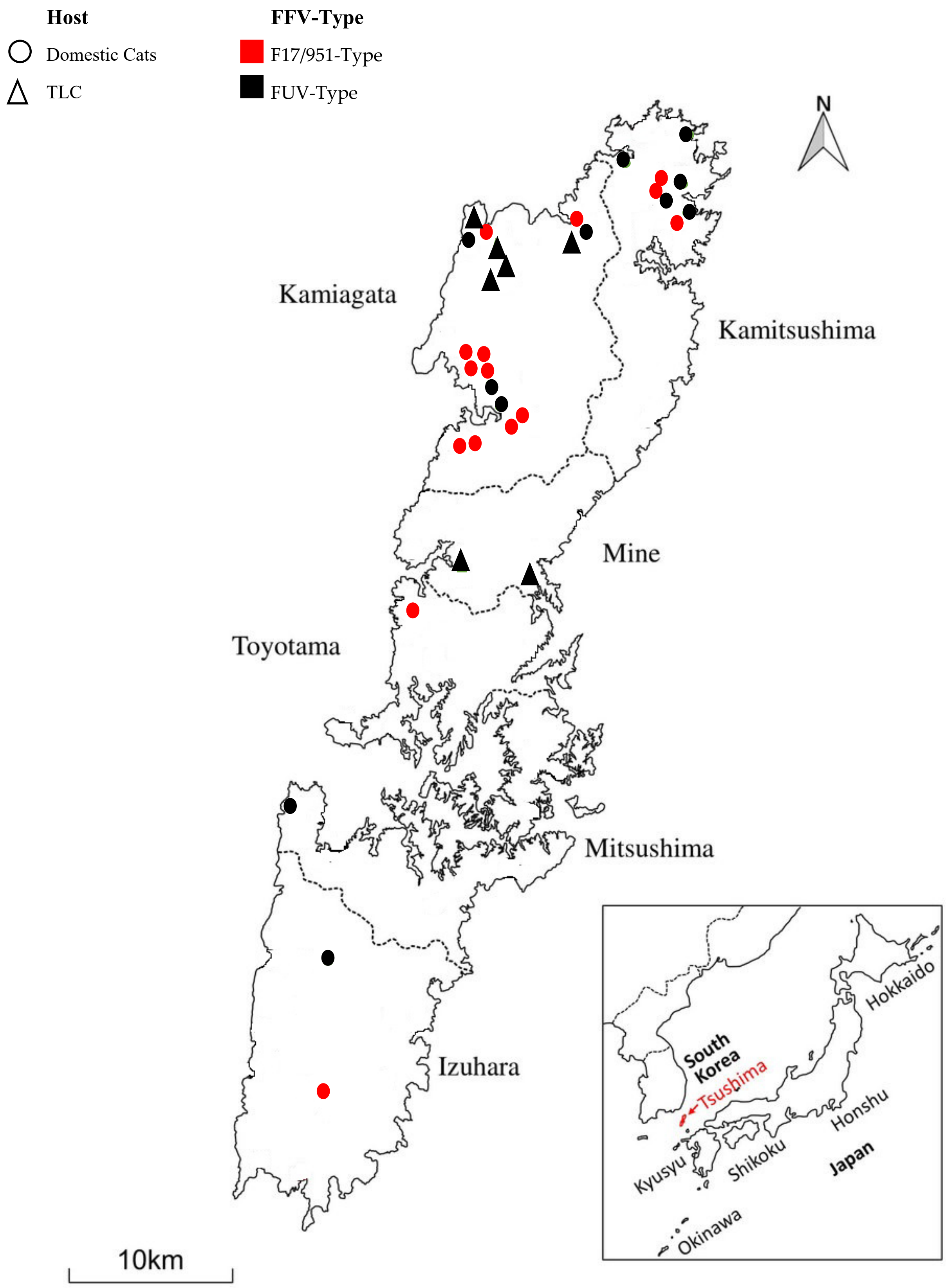

Figure S1. Maps of Tsushima Island and positive sample sites. Cycle indicate domestic cat samples. Triangle indicate TLC samples. Red color indicates F17/951-Type. Black color indicates FUV-Type.
